# Restructuring Reward Mechanisms in Nicotine Addiction: A Pilot fMRI Study of Mindfulness-Oriented Recovery Enhancement for Cigarette Smokers

**DOI:** 10.1155/2017/7018014

**Published:** 2017-03-08

**Authors:** B. Froeliger, A. R. Mathew, P. A. McConnell, C. Eichberg, M. E. Saladin, M. J. Carpenter, E. L. Garland

**Affiliations:** ^1^Department of Neuroscience, Medical University of South Carolina, Charleston, SC, USA; ^2^Department of Psychiatry, Medical University of South Carolina, Charleston, SC, USA; ^3^Hollings Cancer Center, Medical University of South Carolina, Charleston, SC, USA; ^4^Center for Biomedical Imaging, Medical University of South Carolina, Charleston, SC, USA; ^5^Department of Preventive Medicine, Northwestern University Feinberg School of Medicine, Chicago, IL, USA; ^6^Department of Health Sciences and Research, Medical University of South Carolina, Charleston, SC, USA; ^7^College of Social Work, University of Utah, Salt Lake City, UT, USA; ^8^Huntsman Cancer Institute, University of Utah, Salt Lake City, UT, USA

## Abstract

The primary goal of this pilot feasibility study was to examine the effects of Mindfulness-Oriented Recovery Enhancement (MORE), a behavioral treatment grounded in dual-process models derived from cognitive science, on frontostriatal reward processes among cigarette smokers. Healthy adult (*N* = 13; mean (SD) age 49 ± 12.2) smokers provided informed consent to participate in a 10-week study testing MORE versus a comparison group (CG). All participants underwent two fMRI scans: pre-tx and after 8-weeks of MORE. Emotion regulation (ER), smoking cue reactivity (CR), and resting-state functional connectivity (rsFC) were assessed at each fMRI visit; smoking and mood were assessed throughout. As compared to the CG, MORE significantly reduced smoking (*d* = 2.06) and increased positive affect (*d* = 2.02). MORE participants evidenced decreased CR-BOLD response in ventral striatum (VS; *d* = 1.57) and ventral prefrontal cortex (vPFC; *d* = 1.7) and increased positive ER-BOLD in VS (*d*_VS_ = 2.13) and vPFC (*d*_vmPFC_ = 2.66). Importantly, ER was correlated with smoking reduction (*r*'s = .68 to .91) and increased positive affect (*r*'s = .52 to .61). These findings provide preliminary evidence that MORE may facilitate the restructuring of reward processes and play a role in treating the pathophysiology of nicotine addiction.

## 1. Introduction

Cigarette (henceforth nicotine) addiction is a chronic, relapsing brain disorder—resulting in approximately >6 million deaths/year worldwide [1]. Mechanistic research demonstrates that chronic use of addictive substances, including nicotine [2], produces neuroplasticity in mesocorticolimbic circuitry mediating motivation, reward, and conditioned reinforcement [3]. Dual-systems models posit that addiction is subserved by dysregulated interactions between the ventral striatum (VS), a region that codes for the predictive value of a rewarding stimulus (e.g., food, sex, and cigarette) [4], and medial prefrontal cortex (mPFC) that executes attentional control [5], that is, frontostriatal circuitry. Frontostriatal circuitry dysfunction mediates reinstated drug-seeking in animal models [6], and human neuroimaging studies show that individuals with substance-use disorders exhibit weaker resting-state functional connectivity in frontostriatal circuits [7–9]. Within smokers, the magnitude of these deficits is associated with nicotine dependence severity [7], reduced self-reported positive affect, and higher craving and smoking lapses over a 3-day quit period [10]. Thus, dysregulated reward processing is thought to be a primary determinant of addictive behavior, involving a downward shift in the salience of natural reward compared with drug reward [11]. Therefore, targeted interventions that aim to restructure motivation and reward processing around valuation of nondrug related natural rewards and healthy behaviors may promote well-being and confer a therapeutic benefit to quitting smoking [12, 13].

An emerging body of controlled trials indicates that mindfulness-based interventions (MBIs), including Mindfulness-Based Relapse Prevention [14] and Mindfulness-Oriented Recovery Enhancement (MORE) [15], may produce significant therapeutic effects among those struggling with drug addiction, including dependence on alcohol [16], illicit drugs [17], prescription opioids [18], and nicotine [19]. Though prior studies indicate that MBIs are well-tolerated and improve clinical outcomes in persons with substance-use disorders and mindfulness practice is associated with neural response during reward prediction paradigms [20, 21], extant MBIs are not designed to directly restructure reward processes known to predict poor cessation outcomes. Due to their nearly exclusive focus on nonevaluative awareness, existing MBIs do not explicitly teach cognitive reappraisal skills, a therapeutic strategy known to downregulate craving [22]. Nor do MBIs teach techniques to enhance deficiencies in natural reward processing which have been shown to robustly predict smoking relapse [13].

In contrast to other MBIs, MORE integrates mindfulness training with reappraisal and savoring skills designed to decrease valuation of drug reward and amplify natural reward processing, thereby disrupting the cycle of craving, maladaptive affect, and cognition underpinning addictive behavior [15, 23]. Though MORE has been shown to increase autonomic and electrophysiological indices of reward responsivity in chronic pain patients who misuse prescription opioids [24, 25], MORE's effects on reward responsivity in nicotine-dependent smokers and on the frontostriatal circuitry that subserves self-regulated adaptive behavior remain unknown.

The primary aim of the current study was to examine the mechanistic basis of the effects of MORE on restructuring reward processes in nicotine-dependent adult healthy smokers. We sought to test our dual-process model [12] which posits that MORE may restructure reward processes by attenuating drug cue reactivity in the ventral striatum and potentiating prefrontal and striatal responses during positive emotion regulation. Further, we hypothesized that MORE would strengthen resting-state functional connectivity in frontostriatal circuitry involved in appraising the value of reward-predicting stimuli [12]. We secondarily assessed behavioral outcomes including smoking rate and mood.

## 2. Materials and Methods

### 2.1. Participant Characteristics, Recruitment, and Screening Procedures

Participants were recruited from the Charleston metropolitan area through advertisements in regional newspapers and fliers and on Internet sites affiliated with our laboratory. We recruited two separate cohorts: a MORE group and a demographically matched comparison group (CG). Nicotine-dependent adult smokers aged 18 years or higher who reported smoking > 10 cigarettes/day for a minimum of 2 years and had an expired carbon monoxide (CO) concentration of ≥ 10 ppm during a baseline screening visit were included. The following criteria were exclusionary: a past head injury or primary neurological disorder associated with MRI abnormalities; physical or intellectual disability affecting completion of assessments; any contraindication to MRI; use of illicit substances or abuse of prescription medications within the last month; current use of prescription medications that affect the central nervous system (e.g., blood pressure medication) or BOLD response; current or past psychosis; blood alcohol level (BAL) of more than 0.0 on more than one occasion (i.e., during screening); and, among females, a positive pregnancy test (at screening and prior to each fMRI scan).

Participants provided informed consent and then completed an initial screening visit, completed surveys, and trained in a mock scanner. Eligible participants were then invited for the first of two fMRI assessment sessions (fMRI 1). A second and final fMRI assessment was captured at 8 weeks (fMRI 2). All procedures were approved by the MUSC IRB.

### 2.2. Experimental Protocol

#### 2.2.1. MORE Treatment Protocol

The MORE group participated in 10 weekly, 2-hour group sessions led by a Ph.D. level clinical psychologist. MORE sessions involved mindfulness training (including mindful breathing and body scan meditations) to regulate habitual smoking behavior, cognitive reappraisal to decrease negative affect and craving, and savoring [26] to augment natural reward processing and positive emotion. The first three sessions introduced key concepts of mindfulness, cognitive reappraisal, and savoring. Psychoeducation on the nature of craving was introduced at week 4 and followed by sessions focused on coping with stress, mindful attention of the body, and responding to relationship triggers for relapse. Treatment sessions followed the MORE treatment manual [15], which consists of a guide for therapists and handouts for participants. The manual provides (a) theoretical and clinical rationale for each session topic, (b) agendas for each session, (c) scripts for mindfulness exercises, therapeutic techniques, and psychoeducational material, and (d) homework assignments. The authors modified this manual to address issues specific to nicotine addiction, with feedback from two clinical psychologists trained in behavioral smoking cessation interventions. For instance, the sessions were reordered such that session 10, which involves construction of a mindful relapse prevention plan, was moved to session 8 to facilitate the quit-attempt process. The modified MORE treatment manual addresses clinical issues germane to smoking cessation and provides instruction in addressing tardiness and attrition, homework noncompliance, and dealing with barriers to therapy. Quit date was set following week 8. Final sessions addressed the development of a mindful recovery plan and relapse prevention. MORE participants were asked to engage in 15 minutes/day of mindfulness, reappraisal, and savoring practice at home guided by audio CD.

#### 2.2.2. Fidelity and Therapist Adherence/Competence Measures

Therapist adherence and competence was measured with the MORE Fidelity Measure (unpublished). Following Waltz et al. [27] and Carroll et al. [28], items assess therapist behaviors that are unique to MORE, essential to MORE, and compatible with MORE, but neither necessary nor unique to it, and behaviors that are proscribed (to assess protocol violations). Fidelity ratings were used to guide clinical supervision. The last author (the developer of MORE) reviewed audiorecorded sessions to monitor treatment fidelity, and fidelity ratings were used to guide clinical supervision. Any deviations from the treatment manual were communicated weekly prior to the next session during clinical supervision and corrected by the therapist in successive sessions. Minor deviations were observed infrequently, and adherence improved over time; no major deviations were noted.

### 2.3. Comparison Group Protocol

The CG was recruited as a time-control and participated in 2 experimental fMRI sessions that were held 8 weeks apart but did not receive any study treatments.

### 2.4. Self-Report Assessments

#### 2.4.1. Smoking History

Nicotine dependence was measured at baseline using the Fagerström Test for Nicotine Dependence (FTND) [29]. Baseline assessment also captured standard information on smoking (e.g., duration and amount smoked).

#### 2.4.2. Assessment of Craving and Affect

Craving and affect were assessed at baseline and at each of the fMRI visits. Craving and urge to smoke were measured using the modified version of the Shiffman-Jarvik Questionnaire (SJWQ [30]). State-dependent positive affect (PA) and negative affect (NA) were assessed using the 20-item Positive and Negative Affect Schedule (PANAS [31]).

#### 2.4.3. Assessment of Smoking Behavior

Smoking status was serially assessed using Timeline Follow-Back methods and biochemically confirmed via expired breath CO, both at baseline and at each fMRI session (PPM; Vitalograph Breath CO Monitor, Lenexa, KS).

#### 2.4.4. Assessment of Dispositional Mindfulness

Trait mindfulness was assessed at baseline using the Five Facet Mindfulness Questionnaire [32].

### 2.5. Data Analysis

#### 2.5.1. Behavioral Analyses

An independent samples *t*-test was used to assess mean percent cigarette reduction between groups. A mixed ANOVA, controlling for CO value at screening, was performed in SPSS to assess changes in CO from fMRI 1 to fMRI 2 across groups. Two-way repeated-measures ANCOVA were performed independently to assess PA, NA, and craving.

#### 2.5.2. Neuroimaging Data Acquisition, Processing, and Analyses

*Data Acquisition*. Data were collected on a Siemens Magnetom TrioTim 3TMR scanner (Siemens, Erlangen, Germany) with a 32-channel head coil. A 3D, T1-weighted, multiplanar rapid gradient-echo (MPRAGE) sequence was used to acquire high-resolution (1 mm^3^/voxel) structural images. Next, a 6-min, eyes-closed rsFC scan was acquired using an echo-planar gradient-echo pulse sequence (TR = 2000 ms, TE = 30 ms, flip angle = 90°; 36 transverse slices, 3.0 mm thickness, 0.58 ms gap; voxel size was 3.3 mm × 3.3 mm × 3.0 mm), followed a Positive Emotion Regulation Task and then a Cue Reactivity Task.

Structural images were preprocessed using the VBM8 toolbox (http://www.neuro.uni-jena.de/vbm/) for SPM12 (http://www.fil.ion.ucl.ac.uk/spm). Data were preprocessed according to default settings: bias correction; tissue classification/segmentation [33]; partial volume estimation (PVE; [34]); denoising/filtering [33, 35]; warping to the DARTEL IXI-550 template in Montreal Neurologic Institute (MNI) space; and resampling to a (1.5 mm)^3^ voxel size using affine and nonlinear transforms [36]. Forward-deformation fields were calculated from each subject's skull-stripped and rigid-body registered T1 (PVE) image in order to warp functional data into MNI space. Preprocessing of functional data included slice time correction and realignment [37]; motion outlier detection (framewise displacement > 1 mm resting; >4 mm for CR/ER); http://www.nitrc.org/projects/artifact detect) and correction (via interpolation; http://cibsr.stanford.edu/tools/human-brain-project/artrepair-software.html); despiking at 4% of global mean (ER/CR only); coregistration of functional images to PVE image; warping to MNI space using forward deformations and resampling to (1.5 mm)^3^ voxel size; and smoothing with a 10 × 10 × 10 mm FWHM Gaussian filter. Exclusion threshold for rapid motion was 20% of run length, but no subjects exceeded this threshold. Mean volumes corrected did not differ significantly between groups across all tasks and visits (all *p* > 0.10).

*Resting-State*. A 6-min, eyes-closed rsFC scan was acquired using an echo-planar gradient-echo pulse sequence (TR = 2000 ms, TE = 30 ms, flip angle = 90°; 36 transverse slices, 3.0 mm thickness, 0.58 ms gap; voxel size was 3.3 mm × 3.3 mm × 3.0 mm). Preprocessed data were uploaded into the conn14 toolbox (http://www.nitrc.org/projects/conn) for denoising and connectivity analyses. Unsmoothed segmented tissue images, along with functional ROIs constructed from regions of overlap between the CR and ER group × time interactions, were uploaded into the toolbox. Mean time-courses from the unsmoothed BOLD signal from each ROI were characterized with no additional principal components. Confounds (mean white matter (WM) and cerebrospinal fluid (CSF) signal and motion) were regressed out of the mean signal for each ROI. Analysis space was set to match the functional images (i.e., (1.5 mm)^3^) with an explicit mask generated by skull-stripping the DARTEL IXI-550 template image. A band-pass filter of .008 to .09 Hz with despiking performed after confound regression (no detrending) was used. All fMRI data analyses were performed with conn14 toolbox for SPM12. Hypothesis testing was conducted within an a priori restricted search space (DS mask) using seed (i.e., CR/ER overlap region) to voxel bivariate correlations. At the group level, effects of treatment were examined with *t*-tests, correcting for multiple comparisons at the cluster level using Monte Carlo as described previously (*p* < 0.05_voxel_, *K*_*E*_ > 1033). Among significant clusters, weighted means from each ROI were extracted and used for descriptive statistics (mean, SD).

*Positive Emotion Regulation Task. *The study utilizes an event-related ER paradigm that was comprised of a total of 100 trials, each presented for 14 sec, separated by a mean jittered fixation of 4 sec in duration (range 1–7 sec). Each trial was composed of three events. First, an instruction to either “look” or “reappraise” was overlaid onto a positive emotional picture for 2 sec or a neutral picture that contained the look instruction. The so-called “reappraise” strategy instructed participants to imagine experiencing the positive event occurring in the picture and to focus on the enjoyable aspects of the image and their own positive emotional response to the picture. This strategy corresponds to the savoring technique taught in the MORE intervention. Next, the picture was presented alone for 6 sec during which time participants implemented the instructed strategy. Finally, an 8-point rating scale was presented for 6 sec that prompted participants to indicate from 1 (most negative) to 8 (most positive) their current feeling in response to the prior picture. The task was presented in four six-minute runs.* Task Stimuli*. Stimuli included positive and neutral pictures from the International Affective Picture System IAPS [38], selected on the basis of 9-point valence/arousal scales (pos= 7.0, 5.3; neutral = 4–6, <3). Images were only presented once to a participant during the study.

*Cue Reactivity Task. *Participants were scanned while performing a smoking CR task that is a sensitive probe of nicotine dependence [39–42]. The CR task presented alternating blocks of control images (e.g., pencil) (40 sec), followed by a fixation and a craving rating response screen (30 sec), and then smoking-related images (e.g., cigarette) (40 sec) over the course of 8.5 minutes.

For the CR and ER tasks, participant's preprocessed fMRI data from each session were entered into a first-level analysis using the General Linear Model [37] to examine BOLD response during (CR) smoking cue versus neutral blocks and (ER) each of the 3 trial types: view positive, reappraise positive, anda neutral view. CR blocks were modeled using a standard box-car design; ER onsets for each event type were modeled as an impulse at the onset of the event and convolved with a canonical hemodynamic response function. For both tasks, motion was removed through rigid-body rotation and translation and included as covariates, and a high-pass filter (128 seconds; .0078 Hz) was applied to remove slow signal drift. First-level contrast images for the main condition effects were entered into a 2 (group) × 2 (time) repeated-measures ANOVA explicitly masked with a custom whole-brain mask generated by skull-stripping the DARTEL IXI-550 template image. Second-level 2 (group) × 2 (time) random effects analyses were conducted for each task to test for significant group, time, and group × time effects between MORE and control groups, masked by a “dual-systems” (DS) mask made in WFU Pickatlas (http://fmri.wfubmc.edu/software/pickatlas). Briefly, whole-brain significance was defined at *α* = 0.05 (*p* < 0.05; *K*_*E*_ CR > 417; *K*_*E*_ ER > 452), as determined by Monte Carlo simulations (3dClustSim; http://afni.nimh.nih.gov/pub/dist/doc/program_help/3dClustSim.html). Specifically, 3dcalc was used to take the square root of the factorial model's error variance image (ResMS) and 3dFWHMx was used to empirically determine the spatial smoothness of error variance in the model [43]. The calculated FWHM was used in 3dClustSim to estimate the required cluster extent to maintain a 5% type 1 error rate of detecting a “noise-only” cluster. Where significant results were observed, fitted responses adjusted for effect of interest were extracted and inputted into SPSS for descriptive statistics (mean, SD).

## 3. Results

### 3.1. Sample Characteristics

Eighteen participants [MORE *n* = 10, CG *n* = 8] completed fMRI 1, five of whom were lost to follow-up before fMRI 2. The current analyses included only participants with complete data at each session, resulting in a final *N* = 13 (MORE = 7; CG = 6). The full sample characteristics are presented in Table 1.

### 3.2. fMRI Findings

#### 3.2.1. ER Task

**Figure 1:**
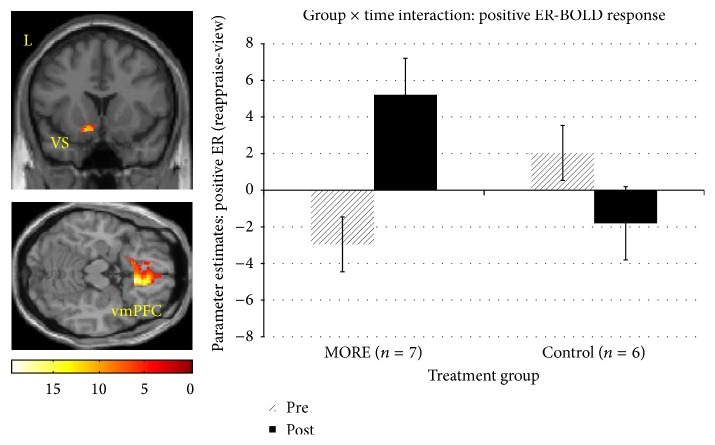
fMRI contrast of the group × time interaction on positive ER-BOLD response. A significant group (MORE, control) × time (Pre, Post) interaction was found in left ventral striatum (VS: −9, 14, −12; *F*(1,22) = 12.4, *d* = 2.13) and right vmPFC (9, 26, −16; *F*'s(1,22) = 19.4, *d* = 2.66, *K*_*e*_ = 1648) (*p*_voxel_ < 0.05, *α* = .05, Monte Carlo). Parameter estimates from the model indicate a relative increase in BOLD response from baseline to 8 weeks post-MORE relative to the control group, who evidenced a decrease in BOLD response.

A significant group × time interaction in right rostral anterior cingulate cortex (rACC) and bilateral ventral striatum was observed for positive ER-BOLD response (Table 2(a); Figure 1). Participants receiving MORE had an increase in BOLD response from Time 1 to Time 2, whereas the CG exhibited the opposite pattern.

#### 3.2.2. CR Task

**Figure 2:**
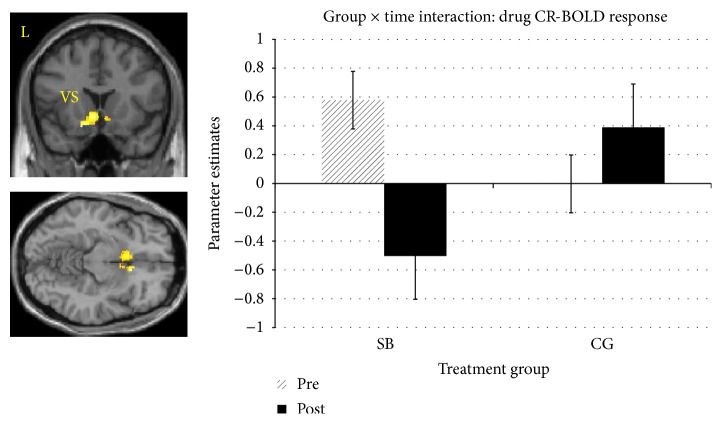
fMRI contrast of the group × time interaction on drug CR-BOLD response. A significant group (MORE, control) × time (Pre, Post) interaction in left ventral striatum (VS: −14, 16, −15) and right vmPFC (10, 20, −10). *F*'s(1,22) = 6.7 to 7.9 *d*'s = 1.57 to 1.7; *K*_*e*_ = 765, (*p*_voxel_ < 0.05, *α* = .05, Monte Carlo). Parameter estimates from the model indicate a relative decrease in BOLD response from baseline to 8 weeks post-MORE relative to the control group, who evidenced an increase in BOLD response.

Another significant group × time interaction in rACC and ventral striatum was revealed for drug CR-BOLD response. Participants receiving MORE had a decrease in BOLD response from Time 1 to Time 2, whereas the CG exhibited an increase across time points (Table 2(b); Figure 2).

#### 3.2.3. Resting-State Functional Connectivity (rsFC)

**Figure 3:**
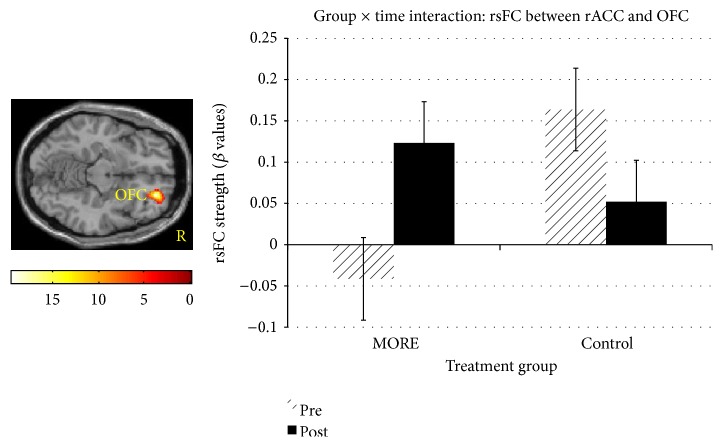
Group (MORE, control) × time (Pre, Post) interaction in right rostral ACC (rACC)-OFC (*x* = 26, *y* = 46, *z* = 12) resting-state functional connectivity, *F*(1,22) = 19.8, *d* = 2.69, *K*_*e*_ = 1330 (*p*_voxel_ < 0.05, *α* = .05, Monte Carlo). The rACC seed was defined by a conjunction mask from the functional ROI clusters from the significant interaction revealed in the positive ER & CR model. Among smokers in the MORE condition, rsFC between rACC and OFC strengthened from baseline to 8 weeks post-MORE, whereas the control group evidenced weaker rsFC between these regions.

A conjunction mask from the overlap between ER and CR task-related findings was generated, revealing a significant cluster in rACC. The rACC cluster then served as the ROI in a seed to voxel rsFC analysis. Results revealed a significant group × time interaction in rsFC between the rACC and orbital frontal cortex. See Figure 3. Among smokers in the MORE condition, rsFC between rACC and OFC strengthened from baseline to 8 weeks post-MORE, whereas the CG evidenced weaker rsFC between these regions.

### 3.3. Correlations between Brain and Behavior (All Correlations Collapsed across Groups)

For positive ER-BOLD response correlates of behavior, time-dependent changes (Time 1 to Time 2) in ER-BOLD response and self-report measures were assessed. Urge to smoke was negatively correlated with ventral-striatal ER-BOLD response (*r* = −.7, *p* = 0.008) and at a trend level with rACC (*r* = −.496, *p* = 0.08). The magnitude of smoking reduction was positively correlated with both ventral-striatal (*r* = .68, *p* = 0.01) and rACC (*r* = .91, *p* < 0.001) ER-BOLD response. PANAS positive affect was positively correlated with ER-BOLD response in rACC (*r* = .614, *p* = 0.025), with a trend observed in ventral striatum (*r* = .52, *p* = 0.07).

For rsFC neural correlates of behavior, the change in strength of rsFC between rACC-OFC, from Time 1 to Time 2, was positively correlated with the magnitude of smoking reduction (*r* = .635, *p* = 0.02), PANAS positive affect (*r* = .773, *p* = 0.002), and positive ER-BOLD response in both the ventral striatum (*r* = .733, *p* = 0.004) and OFC (*r* = .684, *p* = 0.01). Correlations between rACC-OFC rsFC and CR-BOLD response failed to reach significance in the ventral striatum (*r* = −.438, *p* = 0.134) and OFC (*r* = −.495, *p* = 0.08).

### 3.4. Smoking/CO, Affect, and Urge/Craving Outcomes

The change in self-reported average cigarette usage per week from Time 1 to Time 2 was significantly different between groups, as assessed by ANOVA (Table 3(a)). The MORE group (*M* = 66%  ± 10%) reported a greater reduction in weekly cigarette smoking than the CG (*M* = 5%  ± 13%). Self-reported daily cigarette usage at baseline, week 8, and following the quit date (for MORE group) is presented in Table 4 for descriptive purposes. No corresponding group difference in breath CO was observed (Table 3(a)). Analysis of self-reported positive affect identified a group × time interaction (Table 3(b)), which verified that the MORE group exhibited an increase in positive affect from Time 1 (*M* = 30.0 ± 8.0) to Time 2 (*M* = 35.0 ± 9.5), whereas the CG reported decrease from Time 1 (*M* = 30.0 ± 4.0) to Time 2 (*M* = 24.8 ± 5.7). No significant main effects or interaction were identified in the analysis of self-reported negative affect. For urge and craving to smoke, ANOVAs failed to reveal either significant main effects of group and time or an interaction (Table 3(c)).

### 3.5. Emotion Regulation and Cue Reactivity Task Outcomes

Change scores (reappraise – view) were computed for mean self-reported affective rating following each ER task trial and then entered into a 2 (Time: Time 1, Time 2) × 2 (Group: MORE, Control) ANOVA. The main effect of group (*p* = 0.576), time (*p* = 0.057), and group × time interaction (*p* = 0.424) failed to reach significance. The trend towards a significant main effect of time (*d* = 1.28) revealed that participants' self-reported affective ratings following positive ER increased from Time 1 (*M* = .04, SD = .75) to Time 2 (*M* = .57, SD = .75). Change scores (Drug-Control) were computed for mean self-reported craving rating, and then entered into a 2 (Time: Time 1, Time 2) × 2 (Group: MORE, Control) ANOVA. The main effect of group, time, and group × time interaction failed to reach significance (*p*'s > 0.15).

## 4. Discussion

Findings from this pilot study of nicotine-dependent smokers suggest that participation in MORE, a novel mindfulness-based intervention (MBI), was associated with a restructuring of reward responses in the rACC and ventral striatum to natural and drug rewards, the magnitude of which was correlated with reduced smoking behavior and increased positive affect. Study findings provide support for our dual-process model [12] and consistent with our hypotheses regarding frontostriatal mechanisms of mindfulness-centered regulation of addictive behavior.

Following 8 weeks of MORE, participants exhibited enhanced BOLD response in the rACC and ventral striatum during upregulation of positive emotion responses to natural reward stimuli. The positive ER strategy employed during fMRI paralleled the mindful savoring technique taught in the MORE intervention. In this technique, participants are instructed to disengage attention from addiction-related interoceptive and exteroceptive cues and then shift to and sustain attentional focus on the pleasant sensory features of healthful and socially affiliative objects and behaviors. Once attention has been reoriented to the naturally rewarding stimulus, mindfulness is used to metacognitively reflect on positive emotions arising in response to the object or event. This mindful savoring technique involves attention to both the perceptually salient features of the stimulus and its more subtle features, which may enrich the array of sensations and affective experiences to be derived from the savored experience [26, 44].

Deficits in prefrontally mediated neurocognitive and motivational processes are common to substance-use disorders [5]. With regard to nicotine addiction in particular, smokers (as compared to nonsmokers) exhibit dysregulated prefrontal response during cognitive, affective [45], and drug-related cue processing [42]; smoking abstinence produces further disruption in prefrontally mediated neurocognition [46–49]. Further, individuals with a substance-use disorder exhibit potentiated BOLD response in the nucleus accumbens (a striatal region which codes reward related signal [4]) to drug cues [50]. Conversely, the flexible deployment of prefrontal cortex is important in modulating ventral-striatal mediated cigarette craving [51]. Therefore, dysregulated cognitive control over motivational responding, contributing to the downward spiral of drug addiction may be mediated by neuroplasticity in frontostriatal circuitry [6] function shown to underpin craving and positive affect in nicotine-dependent smokers [10].

It is plausible that the effect of MORE on reducing VS reactivity to drug cues may be due to effects on a prefrontal feedback loop required to attenuate VS response to conditioned cues [52]. In other words, increased feedback between frontal cognitive control mechanisms and the VS may help to transition a user from compulsive relapse to regulated use and abstinence [53]. Consistent with this hypothesis, we find that MORE was associated with strengthening in rsFC between the ACC and OFC, regions involved in striatal modulation and appraisal processes, respectively. Taken together, these findings suggest that MORE may potentially restructure bottom-up (striatal) and top-down (ACC/OFC) mechanisms of reward via strengthening communication between these regions.

The potential neural mechanisms identified in the present study might also undergird the increases in autonomic and EEG responses to natural reward stimuli observed in an earlier randomized controlled trial of prescription opioid dependent individuals treated with MORE [24, 25]. Recent analyses of heart rate data from that trial indicate that MORE restructured reward processing by increasing responsiveness to natural reward relative to drug reward, which in turn predicted decreased opioid misuse three months later [54]. Taken prior findings on MORE, with results from the present study, MORE may reverse the allostatic process that underpins addiction by training top-down cognitive control to regulate bottom-up reward processes in service of healthy goal-oriented behavior. Despite these findings, numerous limitations are noted, including a small sample size, baseline differences between groups, and lack of random assignment, and thus they should be interpreted with caution. Therefore, though these novel findings support our a priori hypotheses, larger, randomized controlled neuroimaging studies with long-term follow-ups are needed to replicate the present preliminary findings and ascertain the durability of the effects of MORE on brain reward responses. To our knowledge, the current study is the first to show neuronal changes in reward processes that underlie mindfulness-based treatment, MORE, or otherwise.

## Figures and Tables

**Table 1:** Subject demographics/baseline self-report.

	MORE	Control	Chi^2^/*t*
*Overall sample (N = 13)*	7	6	
% female	0.43	0.14	1.04
Mean age	49.6 (11.3)	48.3 (14.2)	0.18
Years of education	13.7 (1.8)	15.0 (3.3)	0.89
Race			1.04
African Americans	3	1	
Caucasians	4	5	
*Baseline clinical measures*			
Nicotine dependence (FTND)	7.1 (2.1)	6.2 (.8)	1.1
Years smoking	26.9 (11.4)	23.0 (11.3)	0.61
Average daily cigarettes	23.3 (10.9)	21.2 (11.7)	0.34
Carbon monoxide (CO) (screening)	28.3 (10.8)	27.0 (15.6)	0.18
Depressive symptoms (CESD)	17.3 (12.3)	21.7 (14.1)	0.6
Mindfulness (FFMQ)			
Observe	28.7 (4.8)	25.2 (6.3)	1.2
Describe	31.0 (3.9)	28.8 (7.4)	0.68
Awareness	30.4 (6.7)	27.3 (8.2)	0.75
Nonjudgement	28.0 (6.5)	29.2 (6.0)	0.33
Nonreactive	24.1 (4.5)	20.3 (6.5)	1.2

*Note*. Standard deviation reported in parentheses next to mean where applicable.

**Table:** (a) Group × time interaction in positive ER-BOLD response

Side	Regions	Structure	*K*_*e*_ (mm)	MNI	*Z*
				*x*, *y*, *z*	
Right	medial PFC	Rostral ACC	1648	9 26 −16	3.5
Left	Ventral striatum	Caudate		−9 14 −12	2.9
Right	Ventral striatum	Putamen	542	32 10 4	3.1

**Table:** (b) Group × time interaction in CR-BOLD response

Side	Regions	Structure	*K*_*e*_ (mm)	MNI	*Z*
				*x*, *y*, *z*	
Left	Medial PFC	Rostral ACC	765	−14 16 −15	2.3
Left	Ventral Striatum	Caudate		−6 18 −8	2.2
Right	Ventral Striatum	Caudate		10 20 −10	2.1

**Table:** (a) Smoking behavior

	Group		Overall model	
	MORE	Control	*t* (*d*)	*p*
Cigarette reduction	66% (10%)	5% (13)	3.7 (2.06)	0.003

CO (ppm)			*F/t* (*d*)	*p*

Group × time			1.71 (.791)	0.22
Main effect of time	*V*1	*V*2	0.77 (.531)	0.45
MORE	27.3 (6.7)	22.6 (7.5)	2.28 (.253)	0.06
CG	24.3 (7.2)	25.8 (8.1)	0.34 (.055)	0.75

**Table:** (b) Self-reported affect

	Group		Overall model	
	MORE	Control	*F* (*d*)	*p*
PANAS Ppsitive	Group × time		11.1 (2.02)	0.007
Exp *V*1	30.0 (2.5)	30.5 (3.0)		
Exp *V*2	35.0 (2.7)	24.8 (3.3)		
PANAS negative			0.07 (.16)	0.79
Exp *V*1	15.4 (1.9)	13.2 (2.1)		
Exp *V*2	14.4 (1.4)	11.5 (1.5)		

**Table:** (c) State craving on fMRI visit

	Group		Overall model	
	MORE	Control	*F*	*p*
Craving	Group × time		0.07 (.16)	0.8
*V*1	2.7 (.7)	1.3 (.8)		
*V*2	2.4 (.7)	.83 (.7)		
Urge to smoke	Group × time		1.8 (.811)	0.2
*V*1	2.7 (.7)	2.5 (.75)		
*V*2	2.3 (.7)	3.0 (.8)		

**Table 4:** Average number of cigarettes/day over the past week.

Subject	Group	Baseline	Week 8	1 wk. after quitting
MS1	MORE	40.0	33.3	34.1
MS2	MORE	20.9	7.4	0.6
MS3	MORE	14.9	10.6	0.3
MS4	MORE	11.7	8.4	2.7
MS5	MORE	10.4	8.9	2.7
MS6	MORE	11.4	5.6	0.0
MS7	MORE	3.0	2.0	2.7
CGS1	CG	17.1	20.0	na
CGS2	CG	20.0	20.0	na
CGS3	CG	12.0	12.9	na
CGS4	CG	11.4	5.7	na
CGS5	CG	40.0	22.9	na
CGS6	CG	15.0	11.4	na
